# Efficacy of chemotherapy plus gefitinib treatment in advanced non-small-cell lung cancer patients following acquired resistance to gefitinib

**DOI:** 10.3892/mco.2013.156

**Published:** 2013-07-23

**Authors:** ZHENGBO SONG, YIPING ZHANG

**Affiliations:** 1 Department of Chemotherapy, Zhejiang Cancer Hospital, Zhejiang 310022, P.R. China; 2 Key Laboratory Diagnosis and Treatment Technology on Thoracic Oncology, Hangzhou, Zhejiang 310022, P.R. China

**Keywords:** non-small-cell lung cancer, gefitinib, combination, efficacy

## Abstract

Non-small-cell lung cancer (NSCLC) may exhibit oncogene addiction in patients who benefited from prior treatment with epidermal growth factor receptor (EGFR)-tyrosine kinase inhibitors (TKIs). Preclinical data suggested that EGFR addiction persists after the development of TKI resistance, leading many clinicians to continue TKI treatment along with chemotherapy. However, this strategy has not been adequately evaluated in clinical practice. Patients who benefited from gefitinib followed by acquired resistance to this drug were reviewed in the Zhejiang Cancer Hospital. Patients were included if they received chemotherapy and gefitinib following failure of prior gefitinib treatment. A total of 26 patients were included in the present study. Six patients (23.1%) exhibited a partial response (PR), 13 (50%) achieved stable disease (SD) and 7 (26.9%) had progressive disease (PD) during the chemotherapy and gefitinib treatment. The disease control rate (DCR) was 73.1% and the median progression-free survival (PFS) was 4.6 months [95% confidence interval (CI): 3.8–5.4]. The toxicities associated with gefitinib and chemotherapy were generally acceptable. In conclusion, continued concurrent gefitinib and chemotherapy may be a valuable strategy, with acceptable and well-tolerated toxicity. However, this treatment requires further investigation.

## Introduction

Treatment with the epidermal growth factor receptor (EGFR) tyrosine kinase inhibitors (TKIs) gefitinib and erlotinib has led to significant clinical improvement in certain patients with advanced non-small-cell lung cancer (NSCLC), particularly those of Asian descent, non-smokers and those with adenocarcinoma (1–4).

Despite prolonged survival, it should be noted that discontinuation of EGFR inhibition may cause more rapid progression of symptoms and lesions in certain patients, which is referred to as ‘disease flare’ (5). The most likely explanation for this phenomenon is oncogene addiction, which is recognized in several types of cancer. Gastrointestinal stromal tumors have a unique biology and exhibit rapid disease progression when the kinase inhibitor imatinib is removed after prolonged benefit (6). This series describes a similar flare phenomenon in the setting of acquired resistance in EGFR-mutant lung cancer when gefitinib or erlotinib are discontinued due to disease progression.

An effective treatment for patients with disease flare has not yet been established. Preclinical studies indicated that continuous TKI administration may be a valuable strategy. However, available published data on the clinical activity of gefitinib combination chemotherapy following failure of gefitinib are limited. Therefore, the role of combination treatment after gefitinib failure remains remains debatable. This study was retrospectively performed to evaluate the role of combination treatment following gefitinib failure in patients with advanced NSCLC.

## Patients and methods

### Patients

This retrospective study was conducted through a review of medical records of patients with advanced NSCLC who received gefitinib combined with chemotherapy following disease progression due to gefitinib failure, between July, 2010 and June, 2012. The study was approved by the Ethics Committee of the Zhejiang Cancer Hospital. Eligibility criteria included: i) histological or cytological diagnosis of stage IIIb or IV NSCLC; ii) at least one measurable tumor lesion; iii) initial gefitinib treatment for >6 months and acquired resistance to gefitinib according to Jackman’s criteria (7); and iv) discontinuation time between the prior treatment and re-administration of gefitinib of ≤1 week. The characteristics of the study population are shown in Table I.

### Methods

Patients were administered gefitinib orally from day 1 of the first cycle and pemetrexed or docetaxel as an intravenous (i.v.) infusion on day 1. Pemetrexed was administered as a 10-min i.v. infusion and docetaxel 75 mg/m^2^ as a 1-h i.v. infusion once every 3 weeks. Chemotherapy (pemetrexed or docetaxel) was discontinued if no progression occurred at the end of 4 cycles and gefitinib was continuously administered until disease progression.

### Evaluation of response and toxicity

The tumor response was classified in accordance with the Response Evaluation Criteria in Solid Tumors (RECIST) 1.1. The patients were evaluated to determine the stage of their disease prior to treatment initiation and at the time of disease progression or relapse, by computed tomography (CT) of the chest and abdomen and other staging procedures. Adverse events were evaluated according to the Common Terminology Criteria for Adverse Events (CTCAE) 3.0.

### Statistical methods

Kaplan-Meier survival curves were used to estimate overall survival (OS) and progression-free survival (PFS). OS was measured from the first day of combination treatment to the day the patient succumbed or last follow-up. PFS was defined as the interval from the initiation of combination treatment to treatment failure or the date of the last follow-up. All the analyses were performed with SPSS software version 16 (SPSS Inc., Chicago, IL, USA).

## Results

### Patient characteristics

A total of 26 patients (14 males and 12 females) were included in the present study. The median age of the patients was 56 years (range, 42–71 years). The performance status (PS) score was 0–1 in 13 patients (50%) and 2 in the remaining 50%. The majority of tumors (84.6%) were adenocarcinomas with advanced stage at presentation and 34.6% (9/26) of the patients had a history of smoking. The median duration of the initial gefitinib treatment was 9.6 months [95% confidence interval (CI): 7.5–12.0]. Docetaxel was administered to 12 and pemetrexed to 14 patients, concurrently with gefitinib treatment.

### Response data and survival analysis

**Figure 1:**
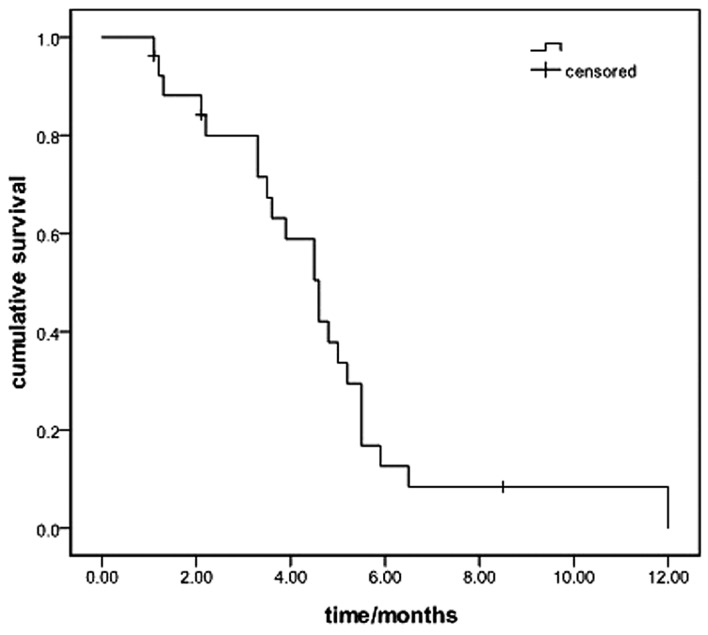
Progression-free survival in the 26 patients.

**Figure 2:**
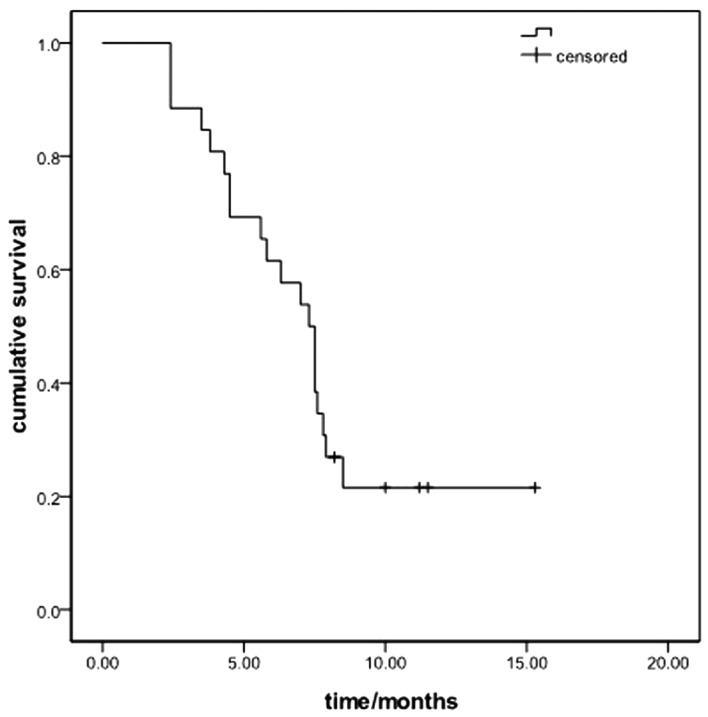
Overall survival in the 26 patients.

The median follow-up period for the 26 patients was 8.0 months (range, 1.0–15 months). Sixteen patients had exhibited a PR and 10 had SD during the prior gefitinib treatment. The response to combination treatment included 6 cases of PR, 13 of SD and 7 of progressive disease (PD), which accounted for a disease control rate (DCR) of 73.1%. The median PFS was 4.6 months (95% CI: 3.8–5.4; Fig. 1). The median OS of the entire patient sample was 7.3 months (95% CI: 6.1–8.5 months) (Fig. 2). Of the 26 patients, 13 underwent analysis of EGFR mutations and 10 were found to harbor activating mutations, including 6 patients with exon 19 deletions and 4 with exon 21 L858R mutations, whereas 3 had a negative mutational status. The median PFS of the 10 patients harboring EGFR mutation was 4.6 months, with 4.2 months for the EGFR wild-type patients (P=0.86).

### Prognostic factors

In the univariate analysis, PS had a statistically significant effect on PFS (Table II). No significant differences in PFS were observed with respect to other factors. The Cox regression model was constructed with the incorporation of age, gender, histological grade, smoking history, chemotherapeutic regimen and PS. PS was identified as the only independent prognostic factor (P=0.043).

### Treatment toxicities

Grade 1/2 skin and hematological toxicities were observed in 13 and 18 patients, respectively. Grade 2 diarrhea developed in 6 patients. Toxicities were considered acceptable, with grade 3/4 skin toxicity in 5 and neutropenia in 9 patients. Two patients had a dosage reduction due to grade 4 neutropenia and 2 patients developed hepatic function abnormalities following gefitinib treatment.

## Discussion

In the present study, the response rate and DCR with gefitinib and chemotherapy following failure of gefitinib treatment were 23.1 and 73.1%, respectively. The median PFS was 4.6 months, which was considered to be favorable compared to previous third- or further-line treatments. The outcome indicated that this treatment was an optimal choice for the patients after failure of gefitinib therapy.

According to the guidelines of the National Comprehensive Cancer Network (8), EGFR-TKIs are recommended as a second- or third-line treatment regimen for patients with NSCLC. However, there were no established treatment protocols for patients following failure of previous gefitinib or erlotinib treatment and discontinuation of EGFR inhibition may cause more rapid progression of symptoms and lesions in certain patients. Chaft *et al*(5) observed a 23% flare rate during the EGFR TKI washout period following disease progression under TKI treatment. Therefore, discontinuation of TKI treatment may not be suitable for patients who benefited from gefitinib or erlotinib.

According to an ASCO 2012 retrospective study, continuation of erlotinib and chemotherapy following failure of erlotinib treatment enhanced the overall response rate (ORR) and achieved a PFS of 4.4 months [Goldberg *et al*(9)]. In the present study the ORR was 23.1%, which was similar to that reported by Goldberg *et al*(9). The outcome indicated that patients may also benefit from the gefitinib combination treatment.

Treatment with gefitinib-pemetrexed or docetaxel was generally well-tolerated and the adverse events (AEs) were similar to those observed in previous studies of each agent alone (10–13). The most common AEs were grade 1/2 skin rash and hematological toxicity.

The present study indicated the efficacy and safety of combined pemetrexed/docetaxel therapy as subsequent treatment in patients with gefitinib-resistant tumors that had exhibited an initial response to gefitinib monotherapy. However, the small sample size of this study may not be sufficient to accurately interpret the study results. Further assessment in a large-scale prospective study is required to obtain definitive evidence. A phase II trial (NCT01707329) has been initiated in our hospital to investigate the efficacy of combination treatment following failure of icotinib, another EGFR-TKI, the efficacy of which was shown to be similar to that of gefitinib in a phase III trial (14).

In conclusion, gefitinib combined with chemotherapy for Chinese patients with advanced NSCLC achieved promising ORR, DCR and PFS, with an acceptable toxicity profile.

## Figures and Tables

**Table I:** Baseline characteristics of the study population (n=26).

Variables	No.	Percentage
Gender		
Male	14	53.8
Female	12	46.2
PS		
0–1	13	50.0
2	13	50.0
Age		
Median	56	
Mean	57	
<65	18	69.2
≥65	8	30.8
Smoking status		
Yes	9	34.6
No	17	65.4
Chemotherapy		
Pemetrexed	14	53.8
Docetaxel	12	46.2
Stage		
IIIb	0	0
IV	26	100
Histology		
Adenocarcinoma	22	84.6
Non-adenocarcinoma	4	15.4
Median duration of prior gefitinib treatment	9.6 months	

PS, performance status.

**Table II:** Univariate analysis of PFS in the 26 patients.

Variables	PFS	95% CI	P-value
Gender			0.013
Male	4.5	2.9–6.3	
Female	4.6	4.2–6.8	
Age (years)			0.57
≥65	4.5	3.2–6.4	
<65	4.8	1.3–7.7	
PS			0.043
0–1	5.5	5.2–5.8	
2	3.3	1.9–4.7	
Chemotherapy			0.86
Pemetrexed	3.9	1.5–6.3	
Docetaxel	4.6	3.9–5.3	
Smoking history			0.40
Yes	4.6	3.4–5.8	
No	5.2	4.0–6.4	
Histology			0.86
Non-adenocarcinoma	3.3	1.1–6.4	
Adenocarcinoma	4.6	4.2–5.0	
Treatment line			0.91
Third-line	4.6	1.1–8.1	
Further-line	4.5	3.3–5.7	

PFS, progression-free survival; CI, confidence interval; PS, performance status.
